# Poster Presentation - REDLARA Congress Andean Region 2024 - Santiago - Chile

**DOI:** 10.5935/1518-0557.20240066

**Published:** 2024

**P-01. Advanced paternal age does not impact the IVF outcomes in donor egg cycles: A single center retrospective analysis**

J. Llanos^1^, N. Sotero^1^, P. Soto^1^, G. Catanzaro^1^, E. Gazzo^1^, F. Peña^1^, R. Dávalos^1^, S. Sessarego^1^, R. Quezada^1^, C. Ascenzo^1^, M. Ascenzo^1^, M. Velit^1^, E. Escudero^1^

^1^ *Inmater Fertility Clinic, Lima, Peru*

**Objective:** To evaluate the impact of advanced paternal age on the in vitro fertilization (IVF) outcomes in donor egg cycles.

**Methods:** Retrospective cohort, data from 140 IVF donor egg cycles using fresh or frozen sperm was evaluated at the Inmater Fertility Clinic in 2022. Excluded criteria were vitrified oocyte or/and donor sperm cycles. All embryos were cultured in an incubator and biopsied on day 5 or 6 when they reached the expanded, hatching or hatched blastocyst stage. Information extracted was oocyte age, biopsy day, morphology based on Gardner criteria, stage classification and Preimplantation Genetic Testing for Aneuploidy (PGT-A). Gardner’s morphology criteria were stratified into four grades: excellent: AA, good: AB, BA, fair: BB, and poor: others. Patients were categorized according to paternal age (group 1 <40 and group 2 ≥40). Differences between groups on IVF outcomes and embryo quality were assessed by the Mann-Whitney U test.

**Results:** A total of 2328 oocytes retrieved were included. After denudation, 2019 metaphase II oocytes were obtained. Group 1 included 55 cycles, and group 2 85 cycles. There were no significant differences between the two groups for oocyte age and seminal parameters (p-value>0.05). As well as not found significant differences between paternal age groups in the IVF outcomes: fertilization rate 2PN, total blastocyst rate, blastocyst utilization rate, top blastocyst rate and euploidy rate (p-value>0.05). Advanced paternal age does not impact embryo quality ≥Grade 4CC in the IVF cycles (p-value>0.05) (Tables 1 and 2).

**Table 1:** Characteristics and semen parameters of the IVF cycles in the different paternal age groups.

Variables	<40(n=55)	≥40(π=85)	p-value
Oocyte age	24.20	24.78	0.125
Paternal age	34.33	45.25	**<0.001**
Semen volume (mL)	1.74	1.53	0.128
Sperm concentration (million/ML)	49.04	49.54	0.941
Sperm progressive motile (%)	36.91	37.62	0.834
Normal semen quality*	32	53	0.752

Data are presented as median or n. Differences between groups were assessed by the Fisher’s exact test and the Mann-Whitney U test.

**Table 2:** Rate of development of transferred embryos.

IVF laboratory data <40 (n = 55)	≥40(π=85)	p-value
Oocytes retrieved 894	1434	
Metaphase II oocytes 755	1264	
Fertilization rate 2PN 79.74 (602)	77.45 (979)	0.241
Total blastocyst rate 63.12 (380)	64.76 (634)	0.517
Day 5 total blastocyst rate 57.81 (348)	60.67 (594)	0.268
Blastocyst utilization rate 58.14 (350)	59.04 (578)	0.752
Day 5 blastocyst utilization rate 44.68 (269)	43.62 (427)	0.715
Blastocyst utilization rate 58.14 (350)	59.04 (578)	0.752
Day 5 blastocyst utilization rate 44.68 (269)	43.62 (427)	0.715
Top blastocyst rate 40.37 (243)	39.73 (389)	0.833
Day 5 top blastocyst rate 30.9 (186)	28.29 (277)	0.280
Blastocyst biopsied 219	433	
Euploidy rate 68.04 (149)	70.21 (304)	0.630

Data are presented as n or % (n). 2PN, two pronuclear. Differences between groups were assessed by the Mann-Whitney U test. Blastocyst utilization: ≥ grade 3CC.

**Conclusion:** This study showed that advanced paternal age when greater than or equal to 40 years old, does not have a significant impact on the IVF outcomes in donor egg cycles.

**P-02. - 2nd Prize - Is the use of hyaluronic acid sperm selection justified in all ICSI cycles? - study of sibling oocytes in own and donor cycles**

J. Portella^1^, L. Bartolo^1^, C. Huamán^1^, Y. Oré^1^, P. Nunja^1^, J. Cárdenas^1^, P. Quispe^1^, A. Seminario^1^

^1^ *Institute of Reproductive Medicine Ricardo Palma Clinic, Lima, Peru*

**Objective:** To compare embryo development when sperm is selected by hyaluronic acid (HA) or polyvinylpyrrolidone (PVP) in intracytoplasmic sperm injection (ICSI) cycles using sibling own oocytes and sibling donor oocytes.

**Methods:** This is a prospective study conducted at the Institute of Reproductive Medicine Ricardo Palma Clinic (Lima, Peru) between June 2023 to March 2024. The fresh or thawed MII oocytes of 67 own oocytes ICSI cycles and 57 donor oocytes ICSI cycles were randomly microinjected by sperm selected in PVP (PVP-ICSI group) or HA-Spermslow™ (HA-ICSI group) assigned in a 1:1 ratio. The inclusion criteria were cycles with at least 4 MII oocytes, ejaculated sperm processed only by gradient density, post-wash progressive motile sperm count ≥1x10^6^/ml, ICSI cycles with or without PGT-A indication. Good-quality (GQ) blastocyst was defined as Gardner Score ≥3AA/3AB/3BA. Mosaic blastocysts were considered containing 30%-80% aneuploid cells found in the trophectoderm biopsy. Fisher exact test was used to analyze the variables. A p-value <0.05 was considered statistically significant.

**Results:** The mean age of the women using their own oocytes were 36.82±3.38 years old (Min: 30yo; Max: 43yo), and recipients women using donor oocytes were 40.95±5.13 years old (Min: 23yo; Max: 51yo). In the groups with own and donor oocytes, the median sperm concentration was 49x10^6^/ml (IQR: 29.75x10^6^ - 67.5x10^6^) and 50x106/ml (IQR: 34x106 – 73.5x10^6^), the progressive motility sperm was 44% (IQR: 35.75% – 51.75%) and 41% (IQR: 32% – 56%), respectively. The results in own oocytes (Table 1) and donor oocytes (Table 2) showed no significant difference between the two groups (PVP-ICSI vs. HAICSI) in terms of fertilization, embryo development and ploidy status.

**Table 1:** Fertilization and embryo development in ICSI cycles with own oocytes inseminated via PVP-ICSI and HA-ICSI (SpermSlow).

Outcome own oocytes	PVP-ICSI	HA-ICSI	p
Fertilization rate (%) (2PN/MII)	73.3% (181/247)	66.9% (166/248)	0.141
Blastulation rate (%)	56.9% (103/181)	49.4% (82/166)	0.164
GQ Blastocyst rate (%)	59.2% (61/103)	45.1% (37/82)	0.075
Blastocyst rate on D5 (%)	44.7% (46/103)	51.2% (42/82)	0.459
Blastocyst rate on D6 (%)	53.4% (55/103)	48.8% (40/82)	0.882
Blastocyst rate on D7 (%)	1.9% (2/103)	0% (0/82)	0.504
Euploid blastocyst rate (%)	43.5% (30/69)	42.9% (21/49)	1
Mosaic blastocyst rate (%)	8.7% (6/69)	14.3% (7/49)	0.381

**Table 2:** Fertilization and embryo development in ICSI cycles with donor oocytes inseminated via PVP-ICSI and HA-ICSI (SpermSlow).

Outcome donor oocytes	PVP-ICSI	HA-ICSI	p
Fertilization rate (%) (2PN/MII)	76.7% (201/262)	75.7% (202/267)	0.838
Blastulation rate (%)	58.2% (117/201)	57.4% (116/202)	0.919
GQ Blastocyst rate (%)	68.4% (80/117)	72.4% (84/116)	0.567
Blastocyst rate on D5 (%)	70.1% (82/117)	63.8% (74/116)	0.332
Blastocyst rate on D6 (%)	27.4% (32/117)	31.9% (37/116)	0.476
Blastocyst rate on D7 (%)	2.6% (3/117)	1.7% (2/116)	1
Euploid blastocyst rate (%)	55.2% (16/29)	57.6% (19/33)	1
Mosaic blastocyst rate (%)	20.7% (6/29)	15.2% (5/33)	0.741

**Conclusion:** In our study, select the sperm by hyaluronic acid in own or donor ICSI cycles no offer benefits on embryo development compared to conventional ICSI. However, this must be extended to a larger study.

**P-03. Prospective comparison of fertilization, cleavage and blastulation rates of human MII sibling oocytes subjected to an ultra-fast vitrification protocol and fresh MII oocytes from oocyte donation program**

P. Ricra^1^, C. Carlos^1^, J. Meza1, W. Montanchez^1^, S. Márquez^1^, P. Pino^1^, L. Noriega-Hoces^2^, L. Noriega-Portella^2^, L Guzman^1^

^1^ *PRANOR Laboratório. Grupo de Reproducción Asistida, Peru.*

^2^ *Clinica Concebir. Lima, Peru.*

**Objective:** To evaluate the survival rate of oocytes undergoing ultra-fast vitrification and compare the fertilization, cleavage and blastulation rates of MII oocytes exposed to ultra-fast vitrification procedure to fresh MII oocytes (control group).

**Methods:** This study included 99 metaphase (MII) oocytes from 8 oocyte donation cycles, where 20 MII oocytes were allocated to ultra-fast vitrification (UFV) protocol and 79 MII fresh oocytes consisted of control group from same ovarian stimulation cycle (sibling oocytes). For ultra-fast vitrification, oocytes were exposed to 100ul-drop of equilibrium solution (ES) for 1 min and then exposed to 100ul-drop of vitrification solution (VS) for 1 min. Oocytes were loaded in cryotop devices (Kitazato®) and plunged into liquid nitrogen. Vitrified oocytes were thawed by conventional warming procedure in TS at 37oC (1 min), then in DS for 3 min, WS for 5 min and WS for min at room temperature (RT). Oocytes were immediately placed in HSA (10%) + Fertilization Total medium for 2 hours. All surviving oocytes underwent intracytoplasmic injection (ICSI) for fertilization. Oocyte survival, fertilization, cleavage, and blastulation rates (BR) were analyzed for fresh and vitrified oocytes.

**Results:** The survival rate of ultra-fast vitrified MII oocytes was 95% (19/20) 2hr post-warming. The fertilization rate post ICSI was 84.2% (16/19) and 68% (54/79) and the cleavage rate was 100% (16/16) and 98% (53/54) for the UFV group and the control group respectively. However, the embryo development was lower than expected when they come from UF vitrified oocytes compared to fresh oocytes (BR: 6% vs. 48%).

**Conclusions:** The results of this study evidence the UFV as a new and faster alternative for oocyte vitrification as this study obtained a high survival rate, showing promising results. Further studies should consider thaw the oocytes with ultra-fast warming protocol (UFW), this might increase the results of embryo development. In addition, it would be interesting to test the embryos obtained with the UFV protocol for euploidy rates and maybe try this protocol with embryos for vitrification and thawing transfers cycles.

**P-04. Analysis of seminal parameters in the first and second semen sample of patients with less than one million of total progressive sperms in their first emission**

M. Arce^1^, T. Castro^1^, J. Gonzáles^1^, M. Sánchez^1^, M. Mestanza^1^, F. Carvallo^1^, A. Franco^1^, J. Pimentel^1^, J. Vásquez^1^

^1^ *Center for Fertility and Assisted Reproduction GERMINAR, Lima, Peru.*

**Objective:** To compare the seminal parameters present in the first and second sperm samples collected same day of patients undergoing an in vitro fertilization procedure, in patients with less than one million total progressive motile sperm in their first emission.

**Methods:** The analysis of the seminal parameters included: volume (mL), concentration (million/mL), motility (%), total number of progressive sperm (TNPS) (mill) from the first and second seminal samples collected on the same day of an in vitro fertilization procedure in 15 patients who attended the GERMINAR Fertility and Assisted Reproduction Center in Lima, Peru, between 2019 and 2023. The study included only patients whose first seminal sample had an TNPS of less than 1 million, regardless of the values reported in their previously performed semen analysis. Statistical analysis used paired Student’s t-test for data meeting the assumptions of normal distribution (Shapiro-Wilk test) and homogeneity of variances (Fisher’s F-test). For data not meeting normality, the non-parametric Wilcoxon test was used.

**Results:** In the analysis of the data, it was found that the second samples showed a trend toward a lower volume compared to the first samples (1.36 mL vs. 1.90 mL); however, no statistical significance was found (p=0.19). In relation to sperm quality, the second emissions had significantly higher sperm concentration (13.59 million/mL vs. 2.57 million/mL) and sperm motility (28.66% vs. 16.14%) compared to the first samples (p<0.05). Finally, the NTMP in the second samples was significantly higher compared to the first samples (4.02 vs. 0.32 million) (p<0.05).

**Conclusion:** In patients who had a first seminal sample with an NTMP of less than 1 million, it was observed that the emission of a second sample improved sperm quality, increasing the concentration, motility, and total number of progressive motile sperm available for assisted reproduction treatment.

**P-05. Asking for a second semen sample on the day of an IVF/ICSI procedure help? Comparing the seminal parameters between a first and second ejaculate**

M. Arce1, T. Castro^1^, J. Gonzáles^1^, M. Sánchez^1^, M. Mestanza^1^, F. Carvallo^1^, A. Franco^1^, J. Pimentel^1^, J. Vásquez^1^

^1^ *Center for Fertility and Assisted Reproduction GERMINAR, Lima, Peru.*

**Objective:** To compare the seminal parameters of a first and second semen sample collected on the same day, from patients undergoing an in vitro fertilization procedure (iVF/ICSI).

**Methods:** Seminal parameters such as volume (mL), concentration (mill/ml), motility (%), and total number of progressive sperm (TNPS) (mill) were compared in semen samples provided by 126 patients who visited the Center for Fertility and Assisted Reproduction GERMINAR for an IVF/ICSI procedures and how were asked to provide 2 semen samples on the same day. Only patients whose their first semen sample had an TNPS lower than their previous sperm analysis by a difference of 20% or more, and if the TNPS was less than or equal to 25 million were included. Statistical analysis used paired Student’s t-test for data meeting the assumptions of normal distribution (Shapiro-Wilk test) and homogeneity of variances (Fisher’s F-test). For data not meeting normality, the non-parametric Wilcoxon test was used.

**Results:** It was observed that the second semen samples had a significantly lower volume compared to the first one: 1.31 ml vs. 1.98 ml (p=2.9e-15). However, regarding sperm quality, the sperm concentration was higher in the second samples than in the first ones (35.24 mill/ml vs. 21.74 mill/ml) (p=6.2e-07), as well as the sperm motility, which was also higher in the second samples compared to the first (35.41% vs. 29.24%) (p=0.0017). Finally, when comparing TNPS between the second and first samples (14.87 mill vs. 9.97 mill), a higher amount was observed in the second samples, but the difference was not statistically significant (p=0.078). Additionally, the TNPS obtained from the mix of the two samples was significantly higher than that obtained from the first semen sample alone (24.85 mill vs. 9.97 mill) (p=0.0017).

**Conclusion:** Asking for a second semen sample on the same day as an IVF/ICSI procedure in patients whose seminal values in the first one do not match their previous sperm analysis and who have an TNPS less than 25 million allowed for an improvement in sample quality and significantly increased the number of progressive motile sperm available.

**P-06. Does pre-implantation genetic testing (PGT-A) improve outcomes for frozen embryo transfers in an egg donation program?**

T. Castro^1^, José Gonzales^1^, Moisés Sánchez^1^, James Mestanza^1^, Flor Carvallo^1^, Jonathan Vásquez^1^

^1^ *Reproductive Medicine Unit, GERMINAR Fertility Center, Lima, Peru.*

**Objective:** To determine whether the transfer of chromosomally normal embryos improves pregnancy and live birth rates compared to the transfer of embryos without genetic testing in an egg donation program.

**Methods:** A cross-sectional retrospective study. 384 transfers of frozen embryos from an egg donation program performed between 2019 and 2023 were evaluated at the GERMINAR Assisted Reproductive Center in Lima-Perú. Only transfers with embryos morphologically classified as A and/or B, according to Gardner’s classification, were included. Patients were divided into 2 groups: Group A with PGT-A (n = 182) and group B without PGT-A (n = 202). Subsequently, only patients who achieved a live birth after one or more transfers were studied: Group A1 with PGT-A (n=94) and Group B1 without PGT-A (n=78). Statistical analysis used Mann-Whitney U and chi-square tests.

**Results:** The average age of the patients was 41.76 and 41.78 years for Groups A and B, respectively (*p*=0.539). On average, 1.17 embryos per procedure were transferred in Group A and 1.63 in group B (*p*=0.000). Group A had a pregnancy rate 64.3% vs. 51.0% (*p*=0.009), and live birth rate of 52.7% *vs.* 39.1% (*p*=0.032), significantly higher compared to Group B. Additionally, in the subgroup of patients who achieved a live birth, it was observed that patients in Group A1 needed a fewer total number of embryos transferred compared to Group B1: 1.48 vs. 2.10 (*p*=0.000); however, no significant difference was found in the total number of transfers performed until achieving a live birth between the two groups: 1.24 *vs.* 1.23 transfers (*p*=0.751). Finally, the multiple pregnancy rate was significantly lower in Group A compared to Group B: 4.9% *vs.* 8.4% (*p*=0.002).

**Conclusions:** The findings showed that in an egg donation program, the transfer of embryos with PGT-A results in better pregnancy and live birth rates, even with a lower number of embryos transferred. Additionally, it results in a lower rate of multiple pregnancies compared to the transfer of embryos without PGT-A.

**P-07. Oral desogestrel to inhibit LH surge in controlled ovarian stimulation for IVF/ICSI cycles**

D. Witker^1^, A. Cortínez^1^, J. F. Alba^1^, M. Luco^1^, J. Guaman^1^, E. Alwane^1^, P. Wells^1^, E. Correa^1^, D. Vantman L^1^, J. A. Pabon^1^, A. Espinoza^1^, F. Sandoval^1^, C. Mella^1^, D. Vantman^1^

^1^ *CER Clinic, Reproductive Medicine, Santiago, Chile.*

**Objective:** To evaluate the efficacy of desogestrel-only oral contraceptive in preventing premature LH surge during controlled ovarian hyperstimulation (COH) in IVF/ICSI cycles and to assess its impact on cycle characteristics and reproductive outcomes.

**Methods:** In this retrospective cohort study conducted from January 1, 2021, to November 30, 2021, 257 patients undergoing IVF/ICSI treatment were included. Participants received a combination of highly purified human menopausal gonadotropin (hMG) or follitropin alfa with a 75 mcg desogestrel-only pill starting on cycle day 1. Ovulation was triggered with a GnRH agonist or hCG when dominant follicles reached maturity. Oocytes were aspirated, fertilized in vitro/ICSI, and viable embryos were cryopreserved for later transfer.

**Results:** The primary outcome was the number of oocytes retrieved. Secondary outcomes included the mean stimulation duration, total gonadotropin dose, number of fertilized oocytes, number of cryopreserved embryos, and incidence of premature LH surge. On average, 11.9 oocytes were retrieved per patient. The mean total gonadotropin dose was 1866.3 IU, and the mean stimulation duration was 10.2 days. The average number of fertilized oocytes was 7.4, and the average number of cryopreserved embryos was 7.2. The incidence of premature LH surge was 0%.

**Conclusion:** Desogestrel effectively prevents premature LH surge during COH in IVF/ICSI cycles, showing promising results in terms of cycle characteristics and reproductive outcomes. Its oral administration is well tolerated and provides a cost-effective alternative to traditional GnRH antagonist protocols, potentially improving accessibility to advanced fertility treatments.

**P-08. 3^rd^ Prize - Is a mosaic embryo an euploid embryo?**

M. Guzmán^1^, M. Mesías^1^, C.Vivar^1^, C. Duarte^1^

^1^ *Centro especializado de Reproducción Asistida NiuVida, Lima, Peru.*

**Objective:** The aim of this study is to evaluate the possibility of transfer a low-grade mosaic embryo (<30%) following the same criteria as in euploid embryos.

**Methods:** This is a retrospective study with a cohort of 38 patients with 38 transfer cycles of 41 mosaic embryos during 2021-2023 at the Centro Especializado de Reproducción Asistida Niu Vida. All embryos were diagnosed using preimplantation genetic aneuploidy analysis (PGT-A) with next-generation sequencing (NGS) following the Preimplantation Genetic Diagnosis International Society (PGDIS) 2019 guidelines. Previously the embryo transfers the patients signed the informed consent and received a genetic counselling. Those embryos were devitrified under the Cryotech protocol and transferred immediately using ultrasound guidance and a Cook catheter. In addition, patients received a supplement of 30 mg of progesterone per day, 25mg of progesterone every 3 days and 6mg of estradiol per day until the pregnancy test. In patients with positive pregnancy results, the supplementation continues until the 10th week of gestation. The follow up of the patients was by phone interview to confirm the development of the pregnancy and birth of the babies.

**Results:** Among the 41 low-grade mosaic embryos transferred 28 (68%) had 1 chromosomal abnormality, 9 (22%) had 2 chromosomal abnormalities and 4 (10%) had 3 chromosomal abnormalities. The transfer of these embryos resulted in 20 clinical pregnancies (52.63%) of which 3 suffered a miscarriage (15%). Of these 17 pregnancies, 15 (83.33%) resulted in live birth and healthy babies. It should be noted that 2 patients could not be followed up.

**Table 1:** Demographic characteristic of patients.

# Of patients	38
Age at the transfer	29-50
Single embryo transfer (SET)	35
Double embryo transfer (DET)	3

**Conclusion:** Our study evidence that low-grade mosaic embryos (<30%) can be transferred as well as euploid embryos. Those embryos can develop into a successful pregnancy with a live birth and healthy babies at home. Based on these results and comparing them with other findings around the world on mosaic embryo transfer, the management of abnormal embryo transfers at our center have changed.

**P-09. 1^st^ Prize - The endometrial functional layer display dissimilarities during natural or hormonal replacement therapy cycles for frozen thawed embryo transfer**

W.A. Palomino^1^, J. Fernandez^1^, A. Carrasquero^1^, F. Irarrazabal^2^, A. Diaz^2^, MC. Johnson^1^

^1^ *Institute for Maternal and Child Research, Faculty of Medicine, University of Chile*

^2^ *Department of Anatomic Pathology San Borja Arriaran Clinical Hospital, Santiago Chile*

**Objective:** To compare the morphologic endometrial structure and progesterone receptor (PGR) expression in functional endometrial compartment during different endometrial preparation schemes for frozen thawed embryo transfer (FET).

**Methods:** Infertile women subjected to assisted reproduction participated in this study. Endometrial biopsies were obtanided from infertile women during mock cycles preceding FET. Samples were obtained during mid luteal phase; 7-8 days after urinary LH surge with confirmed ovulation by ultrasound in natural cycles (NC; n=11) or after 5 days of progesterone supplementation by vaginal route in hormonal replacement therapy (HRT; n=19). Endometrial histology was evaluated according to Noyes criteria. PGR expression was determined by immunohistochemistry using PGR antibody directed to A and B isoforms. PGR expression in epithelial endometrial compartment (EEC) was quantified by Hscore. Comparison of numerical variables were performed by parametric Student T-test test with significance p<0.05

**Results:** No differences in clinical characteristics were found including age, body mass index (BMI) or endometrial thickness during NC or HRT cycles. Endometrial dyssynchrony; more than 3 days of difference between stromal-epithelial compartment development, was observed in 75% of HRT cycles. PGR was localized in the nucleous of both epithelial and stromal cells. PGR Hscore was low in EEC of NC compared to HRT group (0.7±0.1 vs. 2.8±0.6 p=0.001 ).

**Conclusion:** Physiologic PGR down regulation in EEC and synchronic epithelial stromal histologic development are displayed in NC during mid luteal phase. On the contrary, persistent retained PGR in EEC and epithelial-stromal dyssynchrony are depicted in most HRT cycles at the current time employed for FET. The former findings may contribute to understand the embryo implantation and pregnancy development divergences described in natural or HRT cycles for FET.

